# Subchronic GenX Exposure Induces Hepatic Alterations Accompanied by Changes in PPAR-Related Lipid Metabolism and Autophagy-Related Proteins in Adult Male C57BL/6J Mice: Partial Attenuation by Chlorogenic Acid

**DOI:** 10.3390/ph19081164

**Published:** 2026-07-25

**Authors:** Jinjin Zhang, Yu Liu, Yukui Chen, Qi Wang, Xiao-Li Xie

**Affiliations:** 1 Guangdong Provincial Key Laboratory of Tropical Disease Research, Department of Toxicology, School of Public Health, Southern Medical University, No. 1838 North Guangzhou Road, Guangzhou 510515, China; 2 Department of Forensic Pathology, School of Forensic Medicine, Southern Medical University, No. 1838 North Guangzhou Road, Guangzhou 510515, China

**Keywords:** chlorogenic acid, GenX, hepatotoxicity, lipid metabolism, PPAR signaling

## Abstract

**Background:** 2,3,3,3-Tetrafluoro-2-(heptafluoropropoxy)propanoic acid (GenX) is a perfluoroether carboxylic acid that has been detected in drinking water sources. Its potential hepatotoxicity has raised concern, although the associated molecular alterations remain incompletely understood. Chlorogenic acid (CGA), a naturally occurring polyphenol, has been reported to affect oxidative stress and metabolic homeostasis. **Methods:** Adult male C57BL/6J mice were exposed to GenX (2 mg/kg/day) with or without CGA (30 mg/kg/day) by gavage for 12 weeks. AML12 cells were treated with GenX (10–800 μM) for 24 or 48 h to assess cell viability, and intracellular lipid accumulation was evaluated after exposure to 200 μM GenX for 24 h. **Results:** GenX exposure induced hepatomegaly, microvesicular steatosis, inflammatory cell infiltration, and a reduction in hepatic glycogen stores. It also decreased hepatic glutathione concentrations and increased hepatic malondialdehyde concentrations. Serum alanine aminotransferase, aspartate aminotransferase, total cholesterol, and triglyceride levels were elevated. In AML12 cells, GenX increased intracellular lipid accumulation, as assessed by Oil Red O staining. Transcriptomic analysis identified significant enrichment of the peroxisome proliferator-activated receptor (PPAR) signaling pathway. Consistently, GenX altered the expression of genes and proteins involved in lipogenesis, fatty acid uptake, lipid storage, and fatty acid oxidation, suggesting disturbed PPAR-related lipid metabolic regulation. Moreover, the decreased p-mTOR/mTOR ratio, increased LC3-II/I, and overexpression of Beclin1, p62, and inflammatory mediators in the GenX group might suggest changes in autophagy-related proteins and inflammatory response. CGA coadministration partially attenuated several GenX-induced hepatic alterations, including liver enlargement, hepatic lipid accumulation, lipid peroxidation, and changes in selected autophagy- and inflammation-related proteins. **Conclusions:** Subchronic GenX exposure-induced adverse hepatic effects might be associated with disrupted PPAR-related lipid metabolic regulation, oxidative stress, inflammatory responses, and changes in autophagy-related proteins. CGA might exert potential modulatory effects.

## 1. Introduction

Perfluoroalkyl and polyfluoroalkyl substances (PFASs) are synthetic fluorinated compounds with widespread industrial and consumer applications [1]. Perfluorooctanoic acid (PFOA), a prominent member of the PFAS family, has been extensively used in products such as nonstick coatings and food packaging [2]. However, its environmental persistence, bioaccumulative potential, and established toxicities, including hepatotoxicity, developmental toxicity, enterotoxicity, and reproductive toxicity, have raised global concern [3,4,5,6]. Consequently, PFOA is subject to stringent regulations under international agreements such as the Stockholm Convention [7]. Regulatory restrictions on long-chain PFASs have increased the use of replacement compounds, including 2,3,3,3-tetrafluoro-2-(heptafluoropropoxy) propanoic acid, commonly referred to as GenX. Nevertheless, accumulating evidence indicates potential health risks associated with GenX, with its hepatotoxicity being a particular concern. Studies have reported hepatocellular injury, inflammatory changes, oxidative stress, and disturbed lipid metabolism following GenX exposure in mice [8,9,10,11]. These alterations may impair hepatic physiological functions and potentially contribute to the development of liver injury. In 2017, the North Carolina Department of Health and Human Services established a provisional drinking water health goal of 140 parts per trillion (ppt) for GenX [8]. In 2024, the U.S. Environmental Protection Agency established a maximum contaminant level of 10 ppt for GenX in drinking water [12]. These regulatory developments highlight continuing concern regarding exposure to GenX. As the principal organ responsible for xenobiotic metabolism, the liver is particularly susceptible to chemical-induced injury. A more detailed understanding of the hepatic effects of GenX and the biological processes associated with these effects is therefore needed.

Chlorogenic acid (CGA) is a naturally occurring polyphenolic compound found in several plants and plant-derived beverages, including coffee and tea [13]. Its phenolic hydroxyl groups contribute to its free-radical-scavenging and redox-modulating properties [14]. CGA is rapidly metabolized and conjugated in vivo, resulting in low systemic bioavailability of the parent compound. Nevertheless, CGA metabolites and gut microbiota-derived phenolic acids may contribute to its biological effects in the livers [15,16,17]. Previous studies have reported hepatoprotective effects of CGA in several experimental models. In models of alcohol-related liver injury, CGA reduced hepatic reactive oxygen species levels and attenuated steatosis and fibrosis [18]. In mouse models of high-fat diet-induced nonalcoholic fatty liver disease, CGA altered the expression or activity of lipid metabolism-related enzymes and reduced hepatic and serum lipid levels [19]. In models of aflatoxin-induced liver injury, CGA affected B-cell lymphoma 2 (Bcl-2) family proteins, mitochondrial function, and hepatocyte energy metabolism [20]. These findings suggest that CGA may influence several biological processes involved in hepatic injury. However, its potential effects on hepatic injury caused by emerging environmental contaminants such as GenX remain insufficiently characterized.

The present study was designed primarily to investigate the molecular alterations associated with the adverse hepatic effects of subchronic GenX exposure in a murine model. Furthermore, the potential mitigating effects of CGA were investigated. C57BL/6J mice constitute a well-established model for studying diet-induced metabolic disorders. Using male C57BL/6J mice, we assessed the effects of GenX (2 mg/kg/day, dissolved in pure water), administered alone or in combination with CGA (30 mg/kg/day) for 12 weeks, on liver weight, serum biochemical indices, histopathological architecture, and glycogen content. Transcriptomic analysis was conducted on liver tissue to explore the potential molecular mechanisms involved. This study was designed to identify biological pathways potentially involved in GenX-induced hepatic injury and to provide preliminary evidence regarding the modulatory effects of CGA.

## 2. Results

### 2.1. General Observations

**Figure 1:**
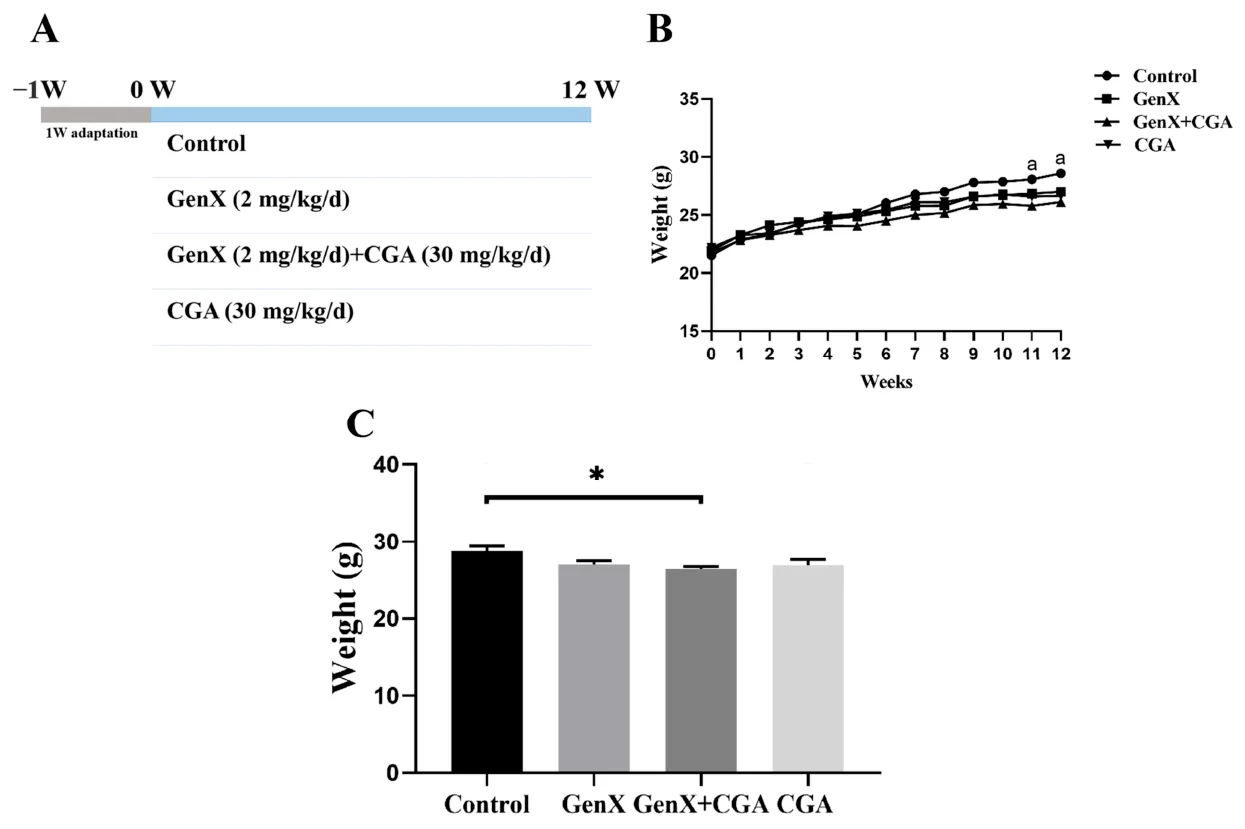
Experimental design and body weight changes in mice exposed to GenX and/or CGA. (**A**) Schematic diagram of the experimental protocol. (**B**) Body weight changes during the 12−week exposure period. a, *p* < 0.05 for GenX + CGA group versus the control group. (**C**) Final body weight at week 12. Control: *n* = 10; GenX: *n* = 12; GenX + CGA: *n* = 12; CGA: *n* = 8. * *p* < 0.05.

The experimental procedure is illustrated in Figure 1A. All the mice survived throughout the experimental period. A significant main effect of time was observed (*p* < 0.001), together with a significant time × treatment interaction (*p* < 0.001), indicating that body weight trajectories differed among the experimental groups during the exposure period (Figure 1B). At week 12, final body weight was 8.05% lower in the GenX + CGA group than in the control group (*p* = 0.023; Figure 1B,C).

### 2.2. CGA Partially Mitigated GenX-Induced Adverse Hepatic Effects

**Figure 2:**
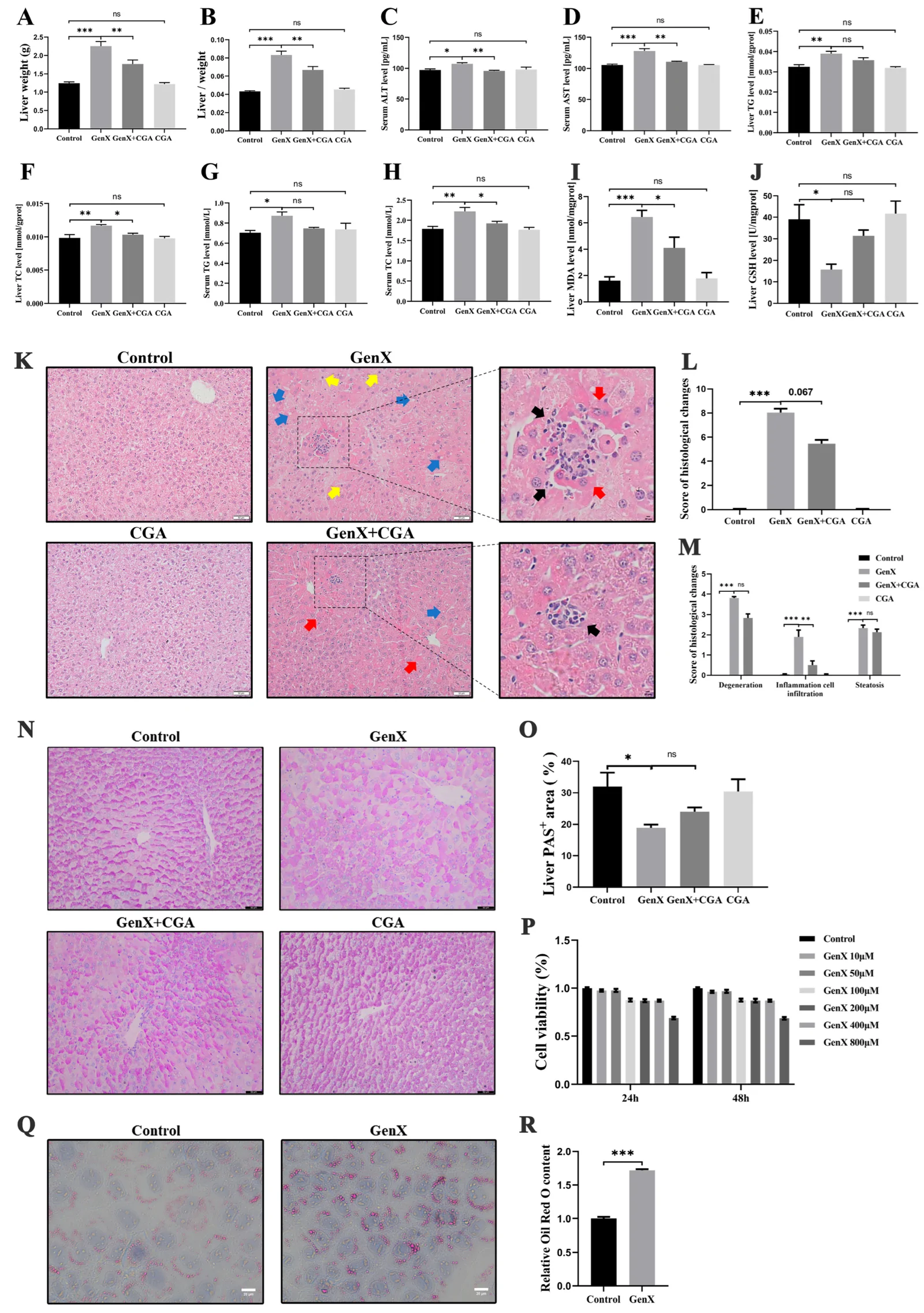
CGA partially attenuated hepatic alterations induced by GenX exposure. (**A**) Absolute liver weight. (**B**) Liver-to-body weight ratio. For panels A and B: control, *n* = 10; GenX, *n* = 12; GenX + CGA, *n* = 12; CGA, *n* = 8. (**C**) Serum ALT levels. (**D**) Serum AST levels. (**E**) Hepatic TG levels. (**F**) Hepatic TC levels. (**G**) Serum TG levels. (**H**) Serum TC levels. (**I**) Hepatic MDA concentrations. (**J**) Hepatic GSH concentrations. For panels (**C**–**J**), *n* = 4 per group. (**K**) Representative H&E-stained liver sections showing eosinophilic degeneration (red arrows), nuclear pyknosis (yellow arrows), lipid vacuolation (blue arrows), and inflammatory cell infiltration (black arrows). Scale bar = 50 μm. (**L**,**M**) Histopathological scores. Control, *n* = 10; GenX, *n* = 12; GenX + CGA, *n* = 12; CGA, *n* = 8. (**N**) Representative PAS-stained liver sections. Scale bar = 50 μm. (**O**) Quantification of glycogen-positive areas in PAS-stained sections; *n* = 5 per group. (**P**) Viability of AML12 cells exposed to GenX. (**Q**) Representative images of Oil Red O-stained AML12 cells. (**R**) Semiquantitative analysis of Oil Red O staining. * *p* < 0.05, ** *p* < 0.01, and *** *p* < 0.001; ns: no significance.

Compared with the control group, the GenX group showed significant increases in absolute liver weight (81.45%, *p* < 0.001; Figure 2A) and the liver-to-body weight ratio (100%, *p* < 0.001; Figure 2B). Serum ALT and AST levels were also elevated by 10.31% (*p* = 0.023; Figure 2C) and 21.14% (*p* < 0.001; Figure 2D), respectively. These findings indicated liver enlargement accompanied by increased biochemical markers of hepatocellular injury. Compared with the GenX group, the GenX + CGA group had lower absolute liver weight (21.33%, *p* = 0.003; Figure 2A), liver-to-body weight ratio (19.58%, *p* = 0.003; Figure 2B), serum ALT level (10.73%, *p* = 0.006; Figure 2C), and serum AST level (13.40%, *p* = 0.001; Figure 2D). These results indicated that CGA coadministration partially attenuated GenX-induced liver enlargement and serum aminotransferase elevations. GenX exposure significantly increased hepatic TG and TC levels by 20.00% (*p* = 0.004) and 18.58% (*p* = 0.003), respectively, compared with the control group (Figure 2E,F). Serum TG and TC levels were also increased by 23.86% (*p* = 0.039) and 23.98% (*p* = 0.002), respectively (Figure 2G,H). Compared with the GenX group, the GenX + CGA group had significantly lower hepatic and serum TC levels, with reductions of 11.67% (*p* = 0.022) and 13.38% (*p* = 0.025), respectively. Hepatic and serum TG levels were lower by 8.21% (*p* = 0.183) and 14.23% (*p* = 0.154), respectively. Moreover, GenX exposure increased hepatic MDA concentrations (*p* < 0.001; Figure 2I) and decreased hepatic GSH concentrations (*p* = 0.023; Figure 2J) relative to the control group. These changes were consistent with increased lipid peroxidation and altered hepatic redox status. Compared with the GenX group, CGA coadministration significantly reduced hepatic MDA concentrations (*p* = 0.046), whereas the increase in GSH concentrations did not reach statistical significance (*p* = 0.154; Figure 2I,J). None of the measured endpoints, including liver weight, the liver-to-body weight ratio, serum ALT and AST levels, hepatic and serum lipid levels, and hepatic MDA and GSH concentrations, differed significantly between the CGA and control groups (Figure 2A–J).

H&E staining showed that hepatocytes in the control group had a regular polygonal appearance, centrally located nuclei, and homogeneous cytoplasm (Figure 2K). In contrast, liver sections from the GenX group showed hepatocellular swelling, microvesicular steatosis, eosinophilic degeneration, nuclear pyknosis or karyolysis, narrowing of the hepatic sinusoids, and inflammatory cell infiltration. Accordingly, the histopathological score was significantly higher in the GenX group than in the control group (*p* < 0.001; Figure 2L,M). Qualitatively, the GenX + CGA group showed less pronounced steatosis, hepatocellular degeneration, sinusoidal narrowing, and inflammatory cell infiltration than the GenX group. However, the reduction in the overall histopathological score did not reach statistical significance (*p* = 0.067; Figure 2K–M). Furthermore, GenX exposure led to a significant reduction in hepatic glycogen-positive area compared with those in the control group (*p* = 0.016, Figure 2N,O). However, compared with those in the GenX group, the hepatic glycogen-positive area in the GenX + CGA group showed a slight increasing trend (*p* = 0.636, Figure 2N,O), suggesting the partial alleviation of CGA, at least in part. Two-way ANOVA identified significant GenX × CGA interactions for selected indicators of liver enlargement, hepatocellular injury, lipid metabolism, and oxidative stress (Table S1). These interaction effects were consistent with a modifying effect of CGA on several GenX-induced hepatic alterations (Table S1).

AML12 cells were exposed to increasing concentrations of GenX for 24 or 48 h to identify an appropriate exposure condition (Figure 2P). On the basis of the cell viability results, exposure to 200 μM GenX for 24 h was selected for the Oil Red O assay. Under this condition, GenX significantly increased intracellular lipid accumulation (*p* < 0.001; Figure 2Q,R).

### 2.3. The PPAR Signaling Pathway Might Involve in GenX-Induced Hepatic Alterations by Transcriptomic Analysis

**Figure 3:**
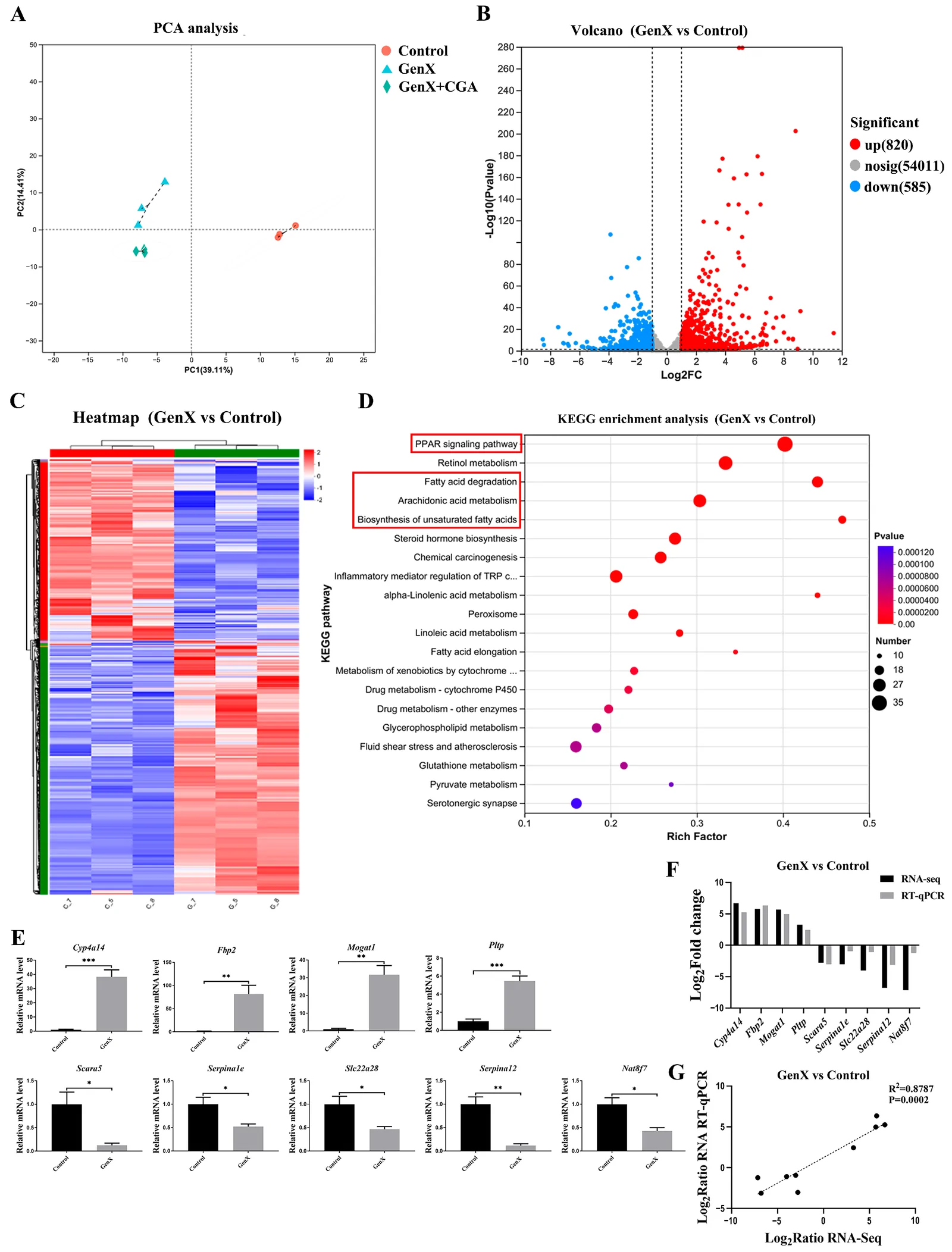
Hepatic transcriptomic analysis suggested the involvement of PPAR signaling in GenX− induced hepatic alterations. (**A**) Principal component analysis of hepatic transcriptomes from the control, GenX, and GenX + CGA groups. (**B**) Volcano plot of hepatic DEGs between the GenX and control groups. (**C**) Heatmap showing hierarchical clustering of DEGs between the GenX and control groups. (**D**) KEGG pathway enrichment analysis of DEGs; the top 20 enriched pathways are shown. (**E**) RT−qPCR analysis of selected DEGs in liver tissues from the GenX and control groups. (**F**) Comparison of log_2_ fold changes obtained by RNA−seq and RT−qPCR for the selected genes. (**G**) Linear regression analysis results. Transcriptomic analysis, *n* = 3 per group; RT−qPCR, *n* = 5 per group. * *p* < 0.05, ** *p* < 0.01, and *** *p* < 0.001.

Principal component analysis of the RNA-seq data showed clear separation among the control, GenX, and GenX + CGA groups, indicating distinct hepatic transcriptomic profiles (Figure 3A). Utilizing stringent criteria (FDR < 0.05 and |log2FC| ≥ 1), 585 downregulated and 820 upregulated differentially expressed genes (DEGs) were identified in the GenX group relative to the control group (Figure 3B). Hierarchical clustering further showed distinct gene expression patterns between the GenX and control groups (Figure 3C). KEGG pathway enrichment analysis identified 71 significantly enriched pathways among the DEGs between the GenX and control groups. The top 20 enriched pathways included PPAR signaling, retinol metabolism, fatty acid degradation, arachidonic acid metabolism, and unsaturated fatty acid biosynthesis (Figure 3D). The enrichment of several lipid metabolism-related pathways suggested that disturbed lipid metabolic regulation was a prominent transcriptomic feature of GenX exposure.

To evaluate the consistency of the RNA-seq findings, nine DEGs were selected for RT-qPCR analysis, including four upregulated and five downregulated genes. The direction of change determined by RT-qPCR was consistent with the RNA-seq results. Specifically, the GenX group showed increased expression of *Cyp4a14* (*p* < 0.001), *Fbp2* (*p* = 0.006), *Mogat1* (*p* = 0.001), and *Pltp* (*p* < 0.001) and decreased expression of *Scara5* (*p* = 0.016), *Serpina1e* (*p* = 0.023), *Slc22a28* (*p* = 0.024), *Serpina12* (*p* = 0.002), and *Nat8f7* (*p* = 0.020) relative to the control group (Figure 3E). Linear regression analysis showed a positive relationship between the log_2_ fold changes determined by RT-qPCR and those obtained by RNA-seq for the selected genes (*p* < 0.001; Figure 3F,G). This agreement supported the consistency of the expression changes measured by the two methods.

### 2.4. Effects of CGA Coadministration on GenX-Induced Transcriptomic and Molecular Alterations

**Figure 4:**
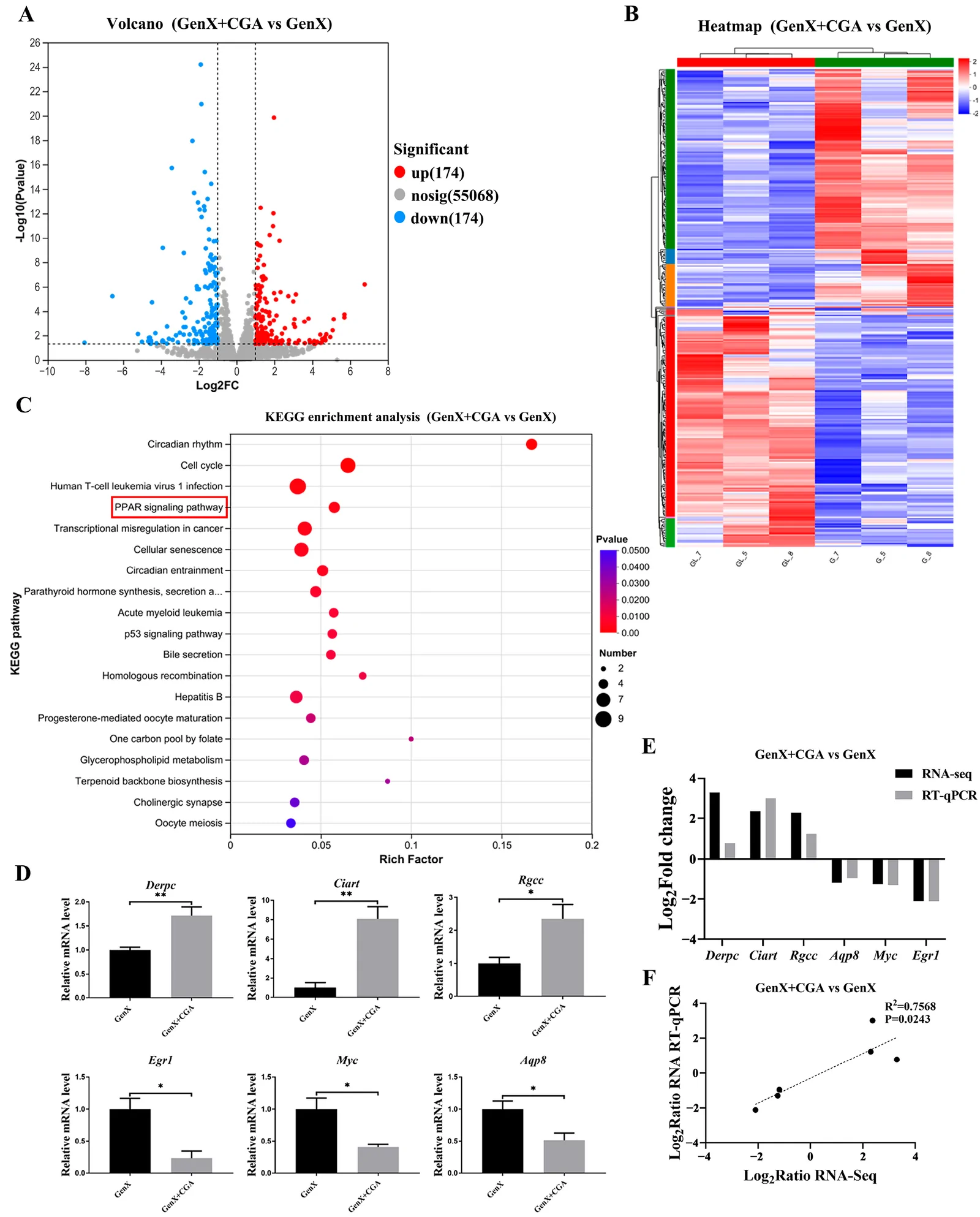
CGA coadministration altered hepatic gene expression in GenX−exposed mice. (**A**) Volcano plot of hepatic DEGs between the GenX + CGA and GenX groups. (**B**) Heatmap showing hierarchical clustering of DEGs between the GenX + CGA and GenX groups. (**C**) KEGG pathway enrichment analysis of DEGs. (**D**) RT−qPCR analysis of selected DEGs in liver tissues from the GenX + CGA and GenX groups. (**E**) Comparison of log_2_ fold changes obtained by RNA−seq and RT−qPCR for the selected genes. (**F**) Linear regression analysis results. Transcriptomic analysis, *n* = 3 per group; RT−qPCR, *n* = 5 per group. * *p* < 0.05 and ** *p* < 0.01.

To characterize the effects of CGA coadministration, hepatic transcriptomes from the GenX + CGA and GenX groups were compared. Using the same DEG thresholds, 174 genes were downregulated and 174 were upregulated in the GenX + CGA group relative to the GenX group (Figure 4A). Hierarchical clustering showed distinct gene expression profiles between the two groups (Figure 4B). KEGG pathway enrichment analysis identified 19 significantly enriched pathways among the DEGs between the GenX + CGA and GenX groups. These pathways included PPAR signaling, bile secretion, and glycerophospholipid metabolism (Figure 4C). The results indicated that CGA coadministration was accompanied by changes in hepatic gene expression, including genes associated with lipid metabolic pathways.

Six DEGs were selected for RT-qPCR analysis to assess consistency with the RNA-seq findings. The selected genes included: *Derpc* (*p* = 0.009), *Ciart* (*p* = 0.002), and *Rgcc* (*p* = 0.029), which were upregulated, and *Egr1* (*p* = 0.010), *Myc* (*p* = 0.016), and *Aqp8* (*p* = 0.047), which were downregulated in the GenX + CGA group relative to the GenX group (Figure 4D). Linear regression analysis showed a positive relationship between the fold changes measured by RT-qPCR and RNA-seq (*p* = 0.024; Figure 4E,F).

PPAR signaling was significantly enriched in both transcriptomic comparisons: GenX versus control and GenX + CGA versus GenX. Previous work by our group also suggested the involvement of autophagy-related processes in GenX-induced liver injury [5]. On the basis of these observations and the lipid metabolic alterations identified in the present study, we further examined selected genes and proteins related to PPAR-associated lipid metabolism, autophagy, and inflammation.

**Figure 5:**
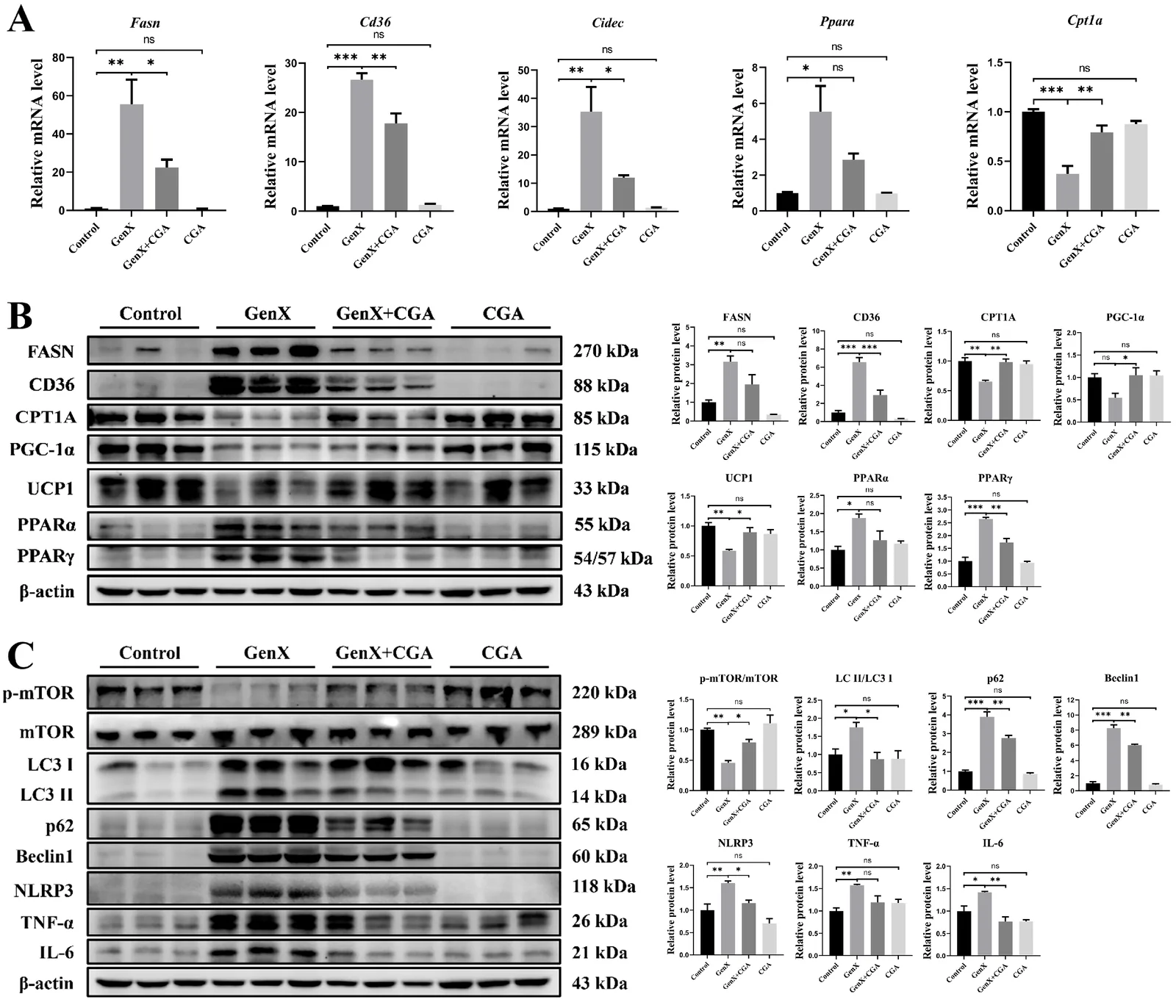
Effects of CGA coadministration on GenX-induced changes in PPAR-related metabolic proteins, autophagy-related proteins, and inflammatory mediators in the mouse liver. (**A**) RT-qPCR analysis of *Fasn*, *Cd36*, *Cidec*, *Ppara*, and *Cpt1a* mRNA expression in the mouse liver. (**B**) Western blot analysis and densitometric quantification of FASN, CD36, CPT1A, PGC-1α, UCP1, PPARα, and PPARγ. (**C**) Western blot analysis and densitometric quantification of the p-mTOR/mTOR ratio, LC3II/I ratio, p62, Beclin1, NLRP3, TNF-α, and IL-6. β-Actin was used as a loading control. *n* = 3 per group. * *p* < 0.05, ** *p* < 0.01, and *** *p* < 0.001, ns: no significance.

GenX exposure increased the hepatic mRNA levels of *Fasn* (*p* = 0.002), *Cd36* (*p* < 0.001), *Cidec* (*p* = 0.002), and *Ppara* (*p* = 0.011), and decreased *Cpt1a* mRNA expression (*p* < 0.001; Figure 5A). In the GenX + CGA group, these alterations were partially attenuated, as evidenced by reduced mRNA levels of *Fasn* (*p* = 0.035), *Cd36* (*p* = 0.004), and *Cidec* (*p* = 0.023), alongside elevated *Cpt1a* transcript levels (*p* = 0.004), relative to the GenX group. *Ppara* mRNA expression showed a nonsignificant downward trend after CGA coadministration (*p* = 0.121; Figure 5A). At the protein level, GenX exposure increased FASN (*p* = 0.005), CD36 (*p* < 0.001), PPARα (*p* = 0.014), and PPARγ (*p* < 0.001) expression and decreased CPT1a (*p* = 0.004) and UCP1 (*p* = 0.006) expression (Figure 5B). PGC-1α expression also showed a downward trend, although the difference was not statistically significant (*p* = 0.078). This expression pattern indicated changes in proteins involved in lipogenesis, fatty acid uptake, fatty acid oxidation, and PPAR-related metabolic regulation. However, these measurements did not directly assess lipid metabolic flux or PPAR transcriptional activity. Compared with the GenX group, the GenX + CGA group showed lower CD36 (*p* < 0.001) and PPARγ (*p* = 0.002) expression and higher CPT1A (*p* = 0.006), PGC-1α (*p* = 0.049), and UCP1 (*p* = 0.032) expression (Figure 5B). FASN and PPARα expression showed nonsignificant downward trends (*p* = 0.093 and *p* = 0.078, respectively). These findings indicated partial normalization of several proteins involved in lipid metabolism following CGA coadministration.

Moreover, GenX exposure significantly decreased the hepatic (*p* = 0.003) and increased the LC3II/I ratio (*p* = 0.047), p62 expression (*p* < 0.001), and Beclin1 expression (*p* < 0.001, Figure 5C). This pattern indicated alterations in proteins involved in autophagy initiation, autophagosome formation, and substrate degradation. Compared with the GenX group, the GenX + CGA group showed an increased p-mTOR/mTOR ratio (*p* = 0.049) and decreased LC3II/I ratio (*p* = 0.022), p62 expression (*p* = 0.004), and Beclin1 expression (*p* = 0.001; Figure 5C). These results indicated that CGA coadministration partially attenuated the GenX-induced changes in the measured autophagy-related proteins. Consistent with the histopathological evidence of inflammatory cell infiltration, GenX exposure increased hepatic NLRP3 (*p* = 0.009), TNF-α (*p* = 0.008), and IL-6 (*p* = 0.024) protein expression (Figure 5C). CGA coadministration reduced NLRP3 (*p* = 0.042) and IL-6 (*p* = 0.002) expression. TNF-α expression also showed a downward trend, but the difference did not reach statistical significance (*p* = 0.066). Two-way ANOVA identified significant GenX × CGA interactions for selected molecular markers related to lipid metabolism, autophagy, and inflammation (Table S1).

These findings suggested that CGA coadministration partially attenuated GenX-induced hepatic lipid accumulation and selected molecular alterations. These effects were accompanied by partial normalization of PPAR-related metabolic markers, autophagy-related proteins, and inflammatory mediators.

## 3. Discussion

A previous study reported that gestational GenX exposure affected both male and female offspring of CD-1 mice. Male offspring showed more pronounced metabolic alterations, whereas female offspring showed greater evidence of liver injury [21]. In the present study, adult male C57BL/6J mice were exposed to GenX for 12 weeks to investigate hepatic metabolic and pathological alterations. GenX exposure induced hepatomegaly, microvesicular steatosis, inflammatory cell infiltration, reduced hepatic glycogen stores, and decreased hepatic GSH concentrations. These changes were accompanied by increased serum ALT, AST, TC, and TG levels and increased hepatic MDA concentrations. The increases in liver weight and the liver-to-body weight ratio were consistent with the marked hepatomegaly observed after GenX exposure. The elevations in serum aminotransferases, together with the histopathological alterations, indicated hepatocellular injury. In addition, the increases in hepatic and serum TC and TG levels and the accumulation of intracellular lipids in AML12 cells supported the presence of disturbed lipid homeostasis. These findings are consistent with previous evidence that the liver is an important target of GenX toxicity. CGA coadministration partially attenuated several GenX-induced hepatic alterations. Specifically, it reduced liver enlargement, serum aminotransferase levels, TC levels, and hepatic MDA concentrations and partially normalized several autophagy- and inflammation-related proteins. Histopathological alterations appeared less pronounced in the GenX + CGA group than in the GenX group; however, the reduction in the overall histopathological score did not reach statistical significance. Body weight was significantly lower in the GenX + CGA group than in the control group at weeks 11 and 12. Because no significant reduction was observed in the CGA group, the lower body weight in the combined-exposure group may reflect an interaction between GenX exposure and CGA coadministration rather than an effect of CGA alone. However, food intake was not monitored. Therefore, possible contributions from altered caloric intake, gastrointestinal effects, or other systemic factors cannot be excluded.

To identify biological processes potentially involved in the hepatic effects of GenX, we performed transcriptomic analysis of mouse liver tissues. KEGG enrichment analysis identified 71 significantly enriched pathways among the DEGs between the GenX and control groups. Several of these pathways were related to lipid metabolism, including PPAR signaling, retinol metabolism, fatty acid degradation, arachidonic acid metabolism, and unsaturated fatty acid biosynthesis. PPAR signaling was also enriched among the DEGs between the GenX + CGA and GenX groups, together with pathways related to bile secretion and glycerophospholipid metabolism. These findings suggested that PPAR-related metabolic regulation may be involved in both the hepatic response to GenX and the changes observed after CGA coadministration. Compared with previous omics-focused studies [22,23], the present study combined transcriptomic analysis with targeted measurements of mRNA and protein expression, hepatic redox-related indices, autophagy-related proteins, and inflammatory mediators. This integrated approach provided convergent evidence for several biological processes associated with GenX-induced hepatic alterations.

The liver is a key organ involved in lipid homeostasis and relies on tightly regulated signaling networks, including the PPAR signaling pathway, to maintain metabolic balance [24]. Human and experimental studies have suggested that PFAS exposure may interfere with metabolic processes associated with metabolic dysfunction-associated steatotic liver disease, potentially with sex-dependent differences in susceptibility [8,21,25]. Epidemiological studies have also reported positive associations between PFAS exposure and dyslipidemia [26,27]. In experimental models, GenX disrupted lipid homeostasis and promoted lipid accumulation in zebrafish [28] and altered hepatic fatty acid and bile acid metabolism in male BALB/c mice [22]. Consistent with these studies, we observed microvesicular steatosis and increased hepatic lipid levels in GenX-exposed C57BL/6J mice.

We therefore examined selected genes and proteins involved in PPAR-related lipid metabolism. GenX increased *Fasn*, *Cd36*, *Cidec*, and *Ppara* mRNA expression. At the protein level, FASN, CD36, PPARα, and PPARγ were increased, whereas CPT1A and UCP1 were decreased. PGC-1α also showed a nonsignificant downward trend. FASN, CD36, and CIDEC are related to lipogenesis, fatty acid uptake, and lipid droplet storage, respectively, whereas CPT1A, PGC-1α, and UCP1 are involved in fatty acid oxidation and energy metabolism. Thus, the observed expression pattern was consistent with disturbed regulation of hepatic lipid metabolism. However, because lipid synthesis and oxidation rates were not directly measured, these data should not be interpreted as direct evidence of altered metabolic flux. CGA coadministration partially reversed several of these expression changes. *Fasn*, *Cd36*, and *Cidec* mRNA expression decreased, whereas *Cpt1a* mRNA expression increased relative to the GenX group. At the protein level, CGA coadministration decreased CD36 and PPARγ expression and increased CPT1A, PGC-1α, and UCP1 expression. FASN and PPARα showed nonsignificant downward trends. These changes were accompanied by reductions in hepatic TC levels and lipid accumulation, suggesting partial attenuation of GenX-induced lipid metabolic disturbances.

PPARs are ligand-activated nuclear receptors that regulate multiple aspects of lipid and energy metabolism [29]. PPARα is particularly important for hepatic fatty acid oxidation, whereas PPARγ contributes to fatty acid uptake, lipid storage, and adipogenic gene expression [30,31]. In several toxicological and metabolic models, pharmacological activation of PPARα increased fatty acid β-oxidation and attenuated oxidative stress, inflammation, and liver injury [32,33]. Previous studies have also reported PPARα activation following GenX exposure in BALB/c mice and humanized mouse models [23,34,35]. However, the biological consequences of PPARα and PPARγ signaling vary according to the ligand, dose, exposure duration, tissue, and experimental model [36,37]. In the present study, both *Ppara* mRNA and PPARα protein expression were increased by GenX exposure, and PPARγ protein expression was also elevated. Increased PPARα abundance, however, does not necessarily indicate increased PPARα transcriptional activity. The concurrent decreases in *Cpt1a* mRNA, CPT1A protein, and UCP1 protein, together with marked hepatic lipid accumulation, suggested that increased PPARα expression was insufficient to maintain lipid oxidative homeostasis under the present exposure conditions. The increase in PPARγ and *Cidec* expression may have been associated with increased lipid storage. This apparently discordant expression pattern may reflect the complex and context-dependent effects of PFASs, including GenX, on PPARα- and PPARγ-related metabolic regulation [38]. Excessive lipid accumulation and mitochondrial stress can contribute to oxidative injury [39,40,41,42]. In the present study, GenX exposure increased hepatic MDA concentrations and decreased GSH concentrations, consistent with increased lipid peroxidation and altered redox homeostasis. Lipid peroxidation may further interfere with mitochondrial function and hepatic lipid metabolism, thereby favoring lipid accumulation [42,43,44]. CGA coadministration reduced hepatic MDA concentrations, suggesting partial attenuation of lipid peroxidation [14,15]. However, because oxidative stress was evaluated using only MDA and GSH, additional measurements would be required to characterize the broader redox response.

Emerging evidence suggests that autophagy-related processes may contribute to PFAS-induced cellular injury [5,45]. mTOR is an important negative regulator of autophagy initiation [46]. Inhibition of mTOR can activate the ULK1–Beclin1 complex, thereby promoting autophagosome formation and LC3-II conversion. As an autophagic cargo receptor, p62 binds LC3-II and ubiquitinated substrates and is generally degraded during effective autophagic flux. Consequently, p62 accumulation can occur when autophagic substrate degradation is impaired [47,48,49,50]. In the present study, GenX exposure decreased the hepatic p-mTOR/mTOR ratio and increased Beclin1 expression and the LC3-II/I ratio. These changes may reflect increased autophagy initiation or autophagosome formation. However, the concurrent increase in p62 may indicate reduced autophagic substrate degradation. GenX exposure also increased hepatic NLRP3, TNF-α, and IL-6 protein expression, consistent with the inflammatory cell infiltration observed by histopathological examination. CGA coadministration reduced NLRP3 and IL-6 expression, whereas TNF-α showed a nonsignificant downward trend. CGA also partially normalized the measured autophagy-related proteins. These findings suggest that CGA attenuated selected inflammatory and autophagy-related changes induced by GenX, although the functional relationships among these processes remain to be determined. CGA has previously been reported to affect oxidative stress and inflammatory signaling in several models [51,52]. In the present study, its effects were not limited to reductions in hepatic MDA and inflammatory mediator expression. CGA also partially normalized several GenX-induced changes in PPAR-related metabolic markers and autophagy-related proteins. These observations raise the possibility that PPAR-related metabolic regulation and autophagy-associated processes contribute to the effects of CGA.

Several limitations should be acknowledged. First, blood and hepatic concentrations of GenX and CGA were not measured. Therefore, we could not determine whether CGA altered the internal exposure, pharmacokinetics, or tissue distribution of GenX. Second, only adult male C57BL/6J mice were examined, which precluded assessment of sex-dependent responses and limited the generalizability of the findings. Third, food intake was not monitored, limiting interpretation of the lower body weight observed in the GenX + CGA group. Fourth, autophagic flux, PPAR transcriptional activity, and lipid metabolic flux were not directly assessed. Future studies should quantify the internal doses of GenX and CGA, include both sexes, and evaluate longer exposure periods to improve relevance to long-term human exposure. Autophagic flux assays, lipid metabolic flux measurements, lipid droplet colocalization studies, and pharmacokinetic analyses are also needed. Functional manipulation of PPARα and PPARγ, using knockdown or pharmacological approaches, would help clarify their roles in GenX-induced hepatic injury and the effects of CGA. Studies using human-derived hepatic models, such as HepaRG cells, would further improve the translational relevance of these findings. Despite the limitations, the present study provides evidence that GenX-induced hepatic injury is accompanied by disturbed lipid metabolic regulation, oxidative stress, inflammatory responses, and changes in autophagy-related proteins. CGA warrants further experimental evaluation as a compound with potential hepatoprotective effects.

## 4. Materials and Methods

### 4.1. Animal Experiments

Six-week-old male C57BL/6J mice were obtained and maintained at the Laboratory Animal Center of Southern Medical University (Guangzhou, China). Male mice were selected because previous evidence suggested that male offspring showed more pronounced metabolic alterations following gestational GenX exposure [21]. male adult mice were chosen to focus on metabolic disorders. All animal procedures complied with the ARRIVE guidelines (Supplementary Material), the Animals (Scientific Procedures) Act 1986, and the National Research Council Guide for the Care and Use of Laboratory Animals. The experimental protocol was approved by the Experimental Animal Ethics Review Committee of Southern Medical University (Approval no. L2021150, approved date: 30 December 2021).

Mice were housed in groups of four per cage in a specific pathogen-free facility maintained at 20–25 °C and 40–60% relative humidity under a 12-h light/12-h dark cycle. Standard rodent chow and sterilized reverse-osmosis deionized (RODI) water were provided ad libitum. The diet supplied approximately 24%, 63%, and 13% of total energy from protein, carbohydrate, and fat, respectively, with an approximate energy density of 3.0 kcal/g. After a 1-week acclimation period, the mice were weighed and allocated to four groups using a body weight-based randomized block design: control (*n* = 10), GenX (*n* = 12), GenX + CGA (*n* = 12), and CGA (*n* = 8). This allocation procedure was used to obtain comparable baseline body weights among the groups. GenX and CGA powders were dissolved directly in sterilized RODI water to prepare working solutions of 0.2 mg/mL and 3 mg/mL, respectively. Mice in the GenX group received GenX (CAS no. 62037-80-3; purity, 95%) by oral gavage at 2 mg/kg/day for 12 weeks. Mice in the GenX + CGA group received the same GenX treatment together with CGA (CAS no. 327-97-9; purity, 98%) at 30 mg/kg/day by oral gavage for 12 weeks. Mice in the control group received an equivalent volume of sterilized RODI water, whereas mice in the CGA group received CGA at 30 mg/kg/day. All treatments were administered once daily. All treatments were administered by one investigator who was blinded to the identity of the numbered treatment solutions. The GenX dose was selected on the basis of previous experimental studies demonstrating hepatic and metabolic effects in mice [21,53,54]. The CGA dose was selected because 30 mg/kg/day has previously been used in studies of experimental liver injury [55,56].

At the end of the 12-week exposure period, mice were deeply anesthetized with 0.5% pentobarbital sodium (80 mg/kg, intraperitoneally). Blood was collected, after which the mice were euthanized by cervical dislocation. Blood samples were centrifuged at 3000 rpm for 15 min at 4 °C, and the serum was stored at −80 °C until biochemical analysis. Liver tissues were rapidly removed and divided into separate portions. One portion was fixed in 4% paraformaldehyde for histopathological examination and glycogen staining. The remaining portions, including samples used for transcriptomic analysis, were snap-frozen in liquid nitrogen and stored at −80 °C. Before outcome assessment, subsets of samples were randomly selected from each group according to the sample requirements of the individual assays.

### 4.2. Cell Culture and Treatment

AML12 cells (CL-0602; Procell Life Science & Technology Co., Ltd., Wuhan, China) were cultured in DMEM/F12 medium (PM150312; Procell) supplemented with 10% fetal bovine serum (A5256701; Gibco, Thermo Fisher Scientific, Waltham, MA, USA), 1% penicillin–streptomycin (GA3502-100ML; Dingguo Biotechnology Co., Ltd., Beijing, China), 0.5% insulin–transferrin–selenium supplement (G4028-10ML; Procell), and 40 ng/mL dexamethasone (PB180607; Procell). Cells were maintained at 37 °C in a humidified atmosphere containing 5% CO_2_ and passaged using 0.25% trypsin (25200056; Gibco). GenX was dissolved in double-distilled water and diluted in culture medium to the required final concentration. Unless otherwise specified for the cell viability assay, cells were exposed to 200 μM GenX for 24 h.

### 4.3. Cell Viability Assay

AML12 cells were seeded in 96-well plates and exposed to GenX at 0, 10, 50, 100, 200, 400, or 800 μM for 24 or 48 h. Cell viability was assessed using a CCK-8 assay kit (CKK8-100T; GBCBIO Technologies Co., Ltd., Guangzhou, China) according to the manufacturer’s instructions. Absorbance was measured at 450 nm. Cell viability was calculated relative to the corresponding vehicle control group and expressed as a percentage.

### 4.4. Serum Biochemical Analysis

Serum ALT and AST were measured using mouse-specific enzyme-linked immunosorbent assay kits (Jiangsu Enzyme Mark Biotech Co., Ltd., Yancheng, China) according to the manufacturer’s instructions (*n* = 4 per group). Each sample was analyzed in a single determination. The reported lower limits of quantification were 2 pg/mL for ALT and 1 pg/mL for AST, and the intra- and interassay coefficients of variation were <15% for both assays.

### 4.5. Hepatic and Serum Lipid Analysis

Hepatic and serum triglyceride (TG) and total cholesterol (TC) levels were determined using commercial enzymatic assay kits (Nanjing Jiancheng Bioengineering Institute, Nanjing, China) according to the manufacturer’s protocols (*n* = 4 per group). Serum was separated by centrifugation at 3000 rpm for 15 min at 4 °C. Liver tissues were homogenized in ice-cold phosphate-buffered saline and centrifuged at 12,000 rpm for 10 min at 4 °C. The resulting supernatants were used for hepatic lipid measurements. Absorbance was measured at 500 nm. Hepatic lipid concentrations were normalized to protein content and expressed as mmol/g protein, whereas serum concentrations were expressed as mmol/L.

### 4.6. Hepatic Oxidative Stress-Related Measurements

Hepatic malondialdehyde (MDA) and glutathione (GSH) concentrations were measured using commercial assay kits (Nanjing Jiancheng Bioengineering Institute, Nanjing, China) according to the manufacturer’s instructions (*n* = 4 per group). Liver tissues were homogenized in ice-cold phosphate-buffered saline and centrifuged at 12,000 rpm for 10 min at 4 °C. The supernatants were collected for biochemical analysis. Absorbance was measured at 532 nm for MDA and 412 nm for GSH. MDA and GSH measurements were normalized to protein content and expressed as nmol/mg protein and U/mg protein, respectively.

### 4.7. Histopathological Analysis

Liver tissues were fixed in a 4% paraformaldehyde for 24 h, dehydrated through a graded ethanol series, embedded in paraffin, and sectioned at a thickness of 3 μm. These sections were stained with hematoxylin and eosin (H&E) and examined using an Olympus BX53 microscope (Olympus, Tokyo, Japan). Histopathological assessment was independently performed by three veterinary pathologists who were blinded to group allocation. At least five randomly selected, nonoverlapping fields were evaluated for each mouse.

Histopathological lesions were semiquantitatively assessed using selected components of the Histological Activity Index and the steatosis component of the NAFLD Activity Score, as described previously [5,57]. Intralobular hepatocellular degeneration and focal necrosis were scored as follows: 0, absent; 1, mild changes involving <1/3 of lobules; 3, moderate changes involving 1/3–2/3 of lobules; and 4, marked changes involving >2/3 of lobules. Portal inflammation was scored as follows: 0, absent; 1, mild inflammatory cell infiltration involving <1/3 of portal tracts; 3, moderate infiltration involving 1/3–2/3 of portal tracts; and 4, marked infiltration involving >2/3 of portal tracts. Steatosis was scored according to the steatosis component of the NAFLD Activity Score as follows: 0, <5%; 1, 5–33%; 2, >33–66%; and 3, >66% of the hepatic parenchyma affected. If the difference between the highest and lowest scores assigned by the three pathologists did not exceed one scoring category, the median score was used. If the difference exceeded one category, the sections were jointly re-evaluated until a consensus score was reached.

### 4.8. Periodic Acid-Schiff (PAS) Staining for Hepatic Glycogen

PAS staining was performed as described in our previous study [5]. Paraffin-embedded liver sections were deparaffinized and rehydrated (*n* = 5 per group). Sections were oxidized in periodic acid for 5–10 min, rinsed, and incubated with Schiff reagent for 15–20 min under protection from light. Nuclei were counterstained with hematoxylin for 3–5 min. Sections were then dehydrated through graded ethanol, cleared in xylene, and mounted with neutral resin. The sections were subjected to gradient alcohol dehydration, xylene clearing, and mounting with neutral resin. PAS-stained sections were examined using an Olympus BX53 microscope (Olympus Corporation, Tokyo, Japan), with glycogen-positive material appearing purplish red. The glycogen-positive area was quantified using ImageJ software (version 1.8.0). For each field, analysis was restricted to a standardized area of 3072 × 2048 pixels to maintain comparability among samples.

### 4.9. Oil Red O Staining and Quantification

Intracellular lipid accumulation in AML12 cells was assessed using an Oil Red O staining kit (MA0120-2; Meilunbio Co., Ltd., Dalian, China). After treatment, cells were fixed with 4% paraformaldehyde (BL539A; Biosharp Biosciences Co., Ltd., Hefei, China) and stained with Oil Red O working solution according to the manufacturer’s instructions. Representative images were acquired using identical microscope settings for all groups. For semiquantitative analysis, intracellular Oil Red O dye was extracted with isopropanol (I811925; Macklin Biochemical Co., Ltd., Shanghai, China), and absorbance was measured at 510 nm. After subtraction of the isopropanol blank, corrected OD_510 values were normalized to the control group and expressed as relative Oil Red O content.

### 4.10. Transcriptomic Analysis

Total RNA was extracted from liver tissues using TRIzol reagent (*n* = 3 per group). RNA integrity was evaluated using a 5300 Bioanalyzer (Agilent Technologies, Santa Clara, CA, USA), and RNA concentration was measured using an ND-2000 spectrophotometer (Thermo Fisher Scientific, Waltham, MA, USA). Samples meeting the following quality criteria were used for library preparation: OD_260/280, 1.8–2.2; OD_260/230, ≥2.0; RNA integrity number, ≥6.5; 28S/18S ratio, ≥1.0; and total RNA amount, >1 μg. RNA purification, reverse transcription, library preparation, and sequencing were performed by Majorbio Biopharm Technology Co., Ltd. (Shanghai, China). Libraries were prepared using the Illumina Stranded mRNA Prep Kit and sequenced on an Illumina NovaSeq 6000 platform (Illumina, Inc., San Diego, CA, USA) with paired-end 150-bp reads. Sequencing generated an average of approximately 40 million clean reads per sample. Raw reads were quality filtered using fastp (version 0.23.2) [58] and aligned to the reference genome using HISAT2 (version 2.2.1) [59]. Transcripts were assembled using StringTie (version 2.2.1) [60], and gene expression was quantified as transcripts per million. DEGs were identified using DESeq2 (version 1.38.3) [61]. Genes with an absolute log_2_ fold change of ≥1 and a false discovery rate-adjusted *p* value of <0.05 were considered differentially expressed. Kyoto Encyclopedia of Genes and Genomes (KEGG) pathway enrichment analysis of the DEGs was performed using the Majorbio Cloud Platform (https://www.majorbio.com/). Enrichment was evaluated using a hypergeometric test, and *p* values were adjusted for multiple comparisons using the Benjamini–Hochberg method. Pathways with an FDR of <0.05 were considered significantly enriched.

### 4.11. Reverse Transcription Quantitative Polymerase Chain Reaction (RT-qPCR)

Total RNA was extracted from liver tissues using TRIzol reagent (*n* = 5 per group), and RNA concentration was measured with a NanoDrop 2000 spectrophotometer (Thermo Fisher Scientific). Complementary DNA was synthesized using PrimeScript RT Master Mix (RR036A; Takara Bio Inc., Kusatsu, Shiga, Japan) according to the manufacturer’s instructions. Quantitative PCR was performed using TB Green Premix Ex Taq GC (RR820A; Takara Bio Inc.) on a LightCycler 96 system (Roche Life Science, Penzberg, Germany). Relative gene expression was calculated using the 2^−ΔΔCt^ method, with β-actin as the reference gene. Primer sequences are provided in Table S2.

### 4.12. Western Blot Analysis

Liver tissues (*n* = 3 per group) were homogenized on ice in radioimmunoprecipitation assay buffer containing protease and phosphatase inhibitors (Dingguo Biotechnology Co., Ltd., Beijing, China). Homogenates were centrifuged at 12,000 rpm for 10 min at 4 °C, and the supernatants were collected as total protein extracts. Protein concentrations were determined using a bicinchoninic acid assay kit (Thermo Fisher Scientific, USA).

Equal amounts of protein were separated by sodium dodecyl sulfate–polyacrylamide gel electrophoresis and transferred to polyvinylidene fluoride membranes. Membranes were blocked with 5% skim milk for 2 h at room temperature and incubated with primary antibodies overnight at 4 °C. After washing, the membranes were incubated with secondary antibodies for 1 h at room temperature. Protein bands were visualized using an enhanced chemiluminescence kit (Guangzhou Meilun Biotechnology Co., Ltd., Guangzhou, China) and an imaging system. Band intensities were quantified using ImageJ software (version 1.8.0). When multiple bands were detected, only the band corresponding to the expected molecular weight of the target protein and showing a consistent pattern across samples was included in the densitometric analysis. Bands at unexpected molecular weights or showing inconsistent background signals were considered nonspecific and excluded. Each target protein was normalized to β-actin detected in the corresponding lane on the same membrane. For analysis of mTOR phosphorylation, p-mTOR and total mTOR were first normalized separately to the corresponding β-actin signals. The p-mTOR/mTOR ratio was then calculated using the β-actin-normalized values.

The primary antibodies were as follows: fatty acid synthase (FASN, sc-48357, 200 µg/mL, Santa Cruz Biotechnology, Inc., Dallas, TX, USA), cluster of differentiation 36 (CD36, sc-7309, 200 µg/mL, Santa Cruz), carnitine O-palmitoyltransferase 1a (CPT1A, YN3388, 1 mg/mL, ImmunoWay Biotechnology, Plano, TX, USA), peroxisome proliferator-activated receptor γ coactivator 1-alpha (PGC-1α, sc-518038, 200 µg/mL, Santa Cruz), uncoupling protein 1 (UCP1, sc-293418, 100 µg/mL, Santa Cruz), peroxisome proliferator-activated receptor alpha (PPARα, sc-398394, 200 µg/mL, Santa Cruz), peroxisome proliferator-activated receptor gamma (PPARγ, sc-7273, 200 µg/mL, Santa Cruz), phosphorylated mammalian target of rapamycin (p-mTOR, sc-293133, 200 µg/mL, Santa Cruz), mammalian target of rapamycin (mTOR, T55306S, 50 µL, Abmart, Shanghai, China), and microtubule-associated protein 1 light chain 3 beta (LC3B, sc-271625, 200 µg/mL, Santa Cruz), sequestosome-1 (p62, sc-25575, 200 µg/mL, Santa Cruz), Beclin1 (sc-48341, 200 µg/mL, Santa Cruz), NLR family pyrin domain containing 3 (NLRP3, TU269693S, 50 µL, Abmart), tumor necrosis factor alpha (TNF-α, sc-52746, 100 µg/mL, Santa Cruz), interleukin-6 (IL-6, sc-57315, 100 µg/mL, Santa Cruz), and β-actin (sc-47778, 200 µg/mL, Santa Cruz). The secondary antibodies were mouse anti-rabbit IgG–HRP (sc-2357, 400 μg/mL; Santa Cruz) and mouse IgGκ binding protein–HRP (sc-516102, 400 μg/mL; Santa Cruz). All primary antibodies were used at a dilution of 1:1000, except β-actin, which was used at 1:2000. Secondary antibodies were used at 1:8000.

### 4.13. Statistical Analysis

Data are presented as the mean ± standard error of the mean. Statistical analyses were performed using SPSS version 25.0. Body weight changes over the 12-week exposure period were analyzed by repeated-measures analysis of variance. Mauchly’s test indicated violation of the sphericity assumption (*p* < 0.001); therefore, Greenhouse–Geisser-corrected results were reported. To characterize group differences at individual time points, one-way analysis of variance was performed separately for each week. Liver weight, the liver-to-body weight ratio, serum ALT and AST levels, hepatic and serum TG and TC levels, hepatic MDA and GSH concentrations, the hepatic glycogen-positive area, four-group RT-qPCR results, and Western blot results were analyzed by two-way analysis of variance. The factors were GenX exposure and CGA treatment. Homogeneity of variance was assessed using Levene’s test. Tukey’s post hoc test was used when variances were homogeneous, whereas Tamhane’s T2 test was used when variances were unequal. Histopathological scores were analyzed using the Kruskal–Wallis test. For analyses involving two groups, including selected RT-qPCR comparisons and Oil Red O quantification, independent-samples *t* tests were used. Relationships between fold changes obtained by RNA-seq and RT-qPCR were evaluated using linear regression analysis. Exact *p* values are provided for key comparisons, and statistical significance is indicated in the corresponding figure legends. A *p* value < 0.05 was considered statistically significant. All analyses were based on biological replicates, with technical replicates averaged before statistical analysis. Unless otherwise stated, n represents the number of biologically independent animals or samples.

## 5. Conclusions

Subchronic GenX exposure induced hepatomegaly, hepatic steatosis, biochemical indicators of hepatocellular injury, lipid peroxidation, and inflammatory alterations in adult male C57BL/6J mice. These effects were accompanied by changes in PPAR-related lipid metabolic markers and autophagy-related proteins. CGA coadministration partially attenuated several GenX-induced hepatic alterations, including liver enlargement, serum aminotransferase elevations, lipid accumulation, hepatic MDA elevation, and changes in selected inflammatory and autophagy-related proteins. Further functional and pharmacokinetic studies are needed to clarify the underlying biological processes and evaluate the translational relevance of CGA.

## Data Availability

The RNA-seq dataset generated in this study has been deposited in the NCBI Sequence Read Archive under BioProject accession no. PRJNA1482915. Additional source data and statistical files are available from the corresponding author upon reasonable request.

## Supplementary Materials

The following supporting information can be downloaded at: https://www.mdpi.com/article/10.3390/ph19081164/s1, Table S1: Main effects and interaction effects from two-way ANOVA; Table S2: Primers for RT-qPCR analysis.
