# Supplementary material for: Subchronic GenX Exposure Induces Hepatic Alterations Accompanied by Changes in PPAR-Related Lipid Metabolism and Autophagy-Related Proteins in Adult Male C57BL/6J Mice: Partial Attenuation by Chlorogenic Acid
Source: Pharmaceuticals (Basel). 2026 Jul 25;19(8):1164. doi: 10.3390/ph19081164 (PMC13516322; doi:10.3390/ph19081164)
Supplement: Supplementary file 1 [file pharmaceuticals-19-01164-s001.zip › Table_S1.pdf]

Table S1. Main effects and interaction effects from two-way ANOVA.

Endpoint	GenX	CGA	GenX*CGA
Weight	0.0556	0.0377	0.2797
Liver Weight	**<0.001**	**0.0139**	**0.0264**
Liver/Weight	**<0.001**	**0.0399**	**0.0091**
ALT	0.0878	**0.0228**	**0.0104**
AST	**<0.001**	**0.0027**	**0.0028**
Liver TG	**<0.001**	0.0997	0.2164
Liver TC	**0.0011**	**0.0248**	**0.0388**
Serum TG	**0.0375**	0.2569	0.0622
Serum TC	**0.0005**	**0.025**	**0.0484**
MDA	**<0.001**	0.0714	**0.0413**
GSH	**0.0047**	0.0832	0.201
HE Score	**<0.001**	**<0.001**	**<0.001**
Liver PAS	**0.0018**	0.5611	0.2754
q-Fasn	**<0.001**	**0.0387**	**0.0412**
q-Cd36	**<0.001**	**0.0075**	**0.0054**
q-Cidec	**<0.001**	**0.0299**	**0.0269**
q-Ppara	**0.0025**	0.1039	0.1085
q-Cpt1a	**<0.001**	**0.0321**	**0.0014**
WB-FASN	**<0.001**	**0.0164**	0.4019
WB-CD36	**<0.001**	**<0.001**	**0.0050**
WB-CPT1A	**0.0120**	**0.0202**	**0.0042**
WB-PGC1a	0.0777	**0.0382**	0.0726
WB-UCP1	**0.0137**	0.1923	**0.0073**
WB-PPARα	**0.0122**	0.1815	**0.0308**
WB-PPARγ	**<0.001**	**0.0028**	**0.0061**
WB-p-mTOR/mTOR	**<0.001**	**0.0173**	0.1588
WB-LC3 II /LC3 I	0.0537	**0.0168**	**0.0474**
WB-p62	**<0.001**	**0.0033**	**0.0118**
WB-Beclin1	**<0.001**	**0.0013**	**0.0034**
WB-NLRP3	**<0.001**	**0.0047**	0.4307
WB-TNF-α	**0.0108**	0.2894	**0.0141**
WB-IL-6	**0.0308**	**<0.001**	**0.0293**
