# Supplementary material for: Subchronic GenX Exposure Induces Hepatic Alterations Accompanied by Changes in PPAR-Related Lipid Metabolism and Autophagy-Related Proteins in Adult Male C57BL/6J Mice: Partial Attenuation by Chlorogenic Acid
Source: Pharmaceuticals (Basel). 2026 Jul 25;19(8):1164. doi: 10.3390/ph19081164 (PMC13516322; doi:10.3390/ph19081164)
Supplement: Supplementary file 1 [file pharmaceuticals-19-01164-s001.zip › Table_S2.pdf]

Table S2. Primers for RT-qPCR analysis.

Gene name	Forward (5′-3′)	Reverse (5′-3′)
Cyp4a14	TCTGGGTTCTTCCAATGGGC	GGACTCGTATATTGCTCCCCG
Mogat1	CTCGTGCAGGTGTGCATTG	GCGTTTTGACAAGACAGATTGG
Fbp2	ACCCTGACCCGTTACGTTATG	ACATTCACGCTCCCCGAAATC
Pltp	CGCAAAGGGCCACTTTTACTA	TAGACCTGTTCGGATGGACAC
Nat8f7	CAGGAGTGTGGTGGATTTGTT	GACCCCGAGTAAGAGCAGG
Serpina12	TTGCTCGACACAACATGGAAT	ACGTCCCAGTTTGACATCTCT
Slc22a28	TTAAGGGCAACAGGTATGGGA	GGGGCCAGGAATAATCCACT
Scara5	GAGAGCTGAATGTTGCCGAG	AAAACAGTTGGCAGACGGTC
Serpina1e	TAGGGAGCAAGGGTGACACTC	ACTGTCTGGTCTGTTGAGGGT
Derpc	GCCATGCTTCGGTAACTCTG	AGTCCCAGAAGAAAGGAGCC
Ciart	CTGAACGGACTCAAGATGGGT	ACCTCCTGAGGATGACTTCTG
Rgcc	GAGCGCCACTTCCACTATGAG	GGAGAGGAGTTGGTTGGAGAA
Egr1	TCGGCTCCTTTCCTCACTCA	CTCATAGGGTTGTTCGCTCGG
Myc	ATGCCCCTCAACGTGAACTTC	CGCAACATAGGATGGAGAGCA
Aqp8	ACACCAATGTGTAGTATGGACCT	TGACCGATAGACATCCGATGAAG
FASN	AGGTGGTGATAGCCGGTATGT	TGGGTAATCCATAGAGCCCAG
CD36	AGATGACGTGGCAAAGAACAG	CCTTGGCTAGATAACGAACTCTG
PPARα	TACTGCCGTTTTCACAAGTGC	AGGTCGTGTTCACAGGTAAGA
Cpt1a	CTATGCGCTACTCGCTGAAGG	GGCTTTCGACCCGAGAAGA
Cidec	ATGGACTACGCCATGAAGTCT	CGGTGCTAACACGACAGGG
β-actin	CGGCTGTATTCCCTCCATCG	CCAGTTGGTAACAATGCCATGT
